# A continuum description of the buckling of a line of spheres in a transverse harmonic confining potential

**DOI:** 10.1098/rsos.230293

**Published:** 2023-07-12

**Authors:** S. Hutzler, J. Ryan-Purcell, A. Mughal, D. Weaire

**Affiliations:** ^1^ School of Physics, Trinity College Dublin, The University of Dublin, Dublin, Republic of Ireland; ^2^ Department of Mathematics, Aberystwyth University, Penglais, Aberystwyth, Ceredigion, Wales SY23 3BZ, UK

**Keywords:** one-dimensional colloidal chains, buckling, Jacobi functions, Whittaker functions, Airy functions

## Abstract

A line of contacting hard spheres, placed in a transverse confining potential, buckles under compression or when tilted away from the horizontal, once a critical tilt angle is exceeded. This interesting nonlinear problem is enriched by the combined application of both compression and tilt. In a continuous formulation, the profile of transverse sphere displacement is well described by numerical solutions of a second-order differential equation (provided that buckling is not of large amplitude). Here we provide a detailed discussion of these solutions, which are approximated by analytic expressions in terms of Jacobi, Whittaker and Airy functions. The analysis in terms of Whittaker functions yields an exact result for the critical tilt for buckling without compression.

## 1. Introduction

**Figure 1.**
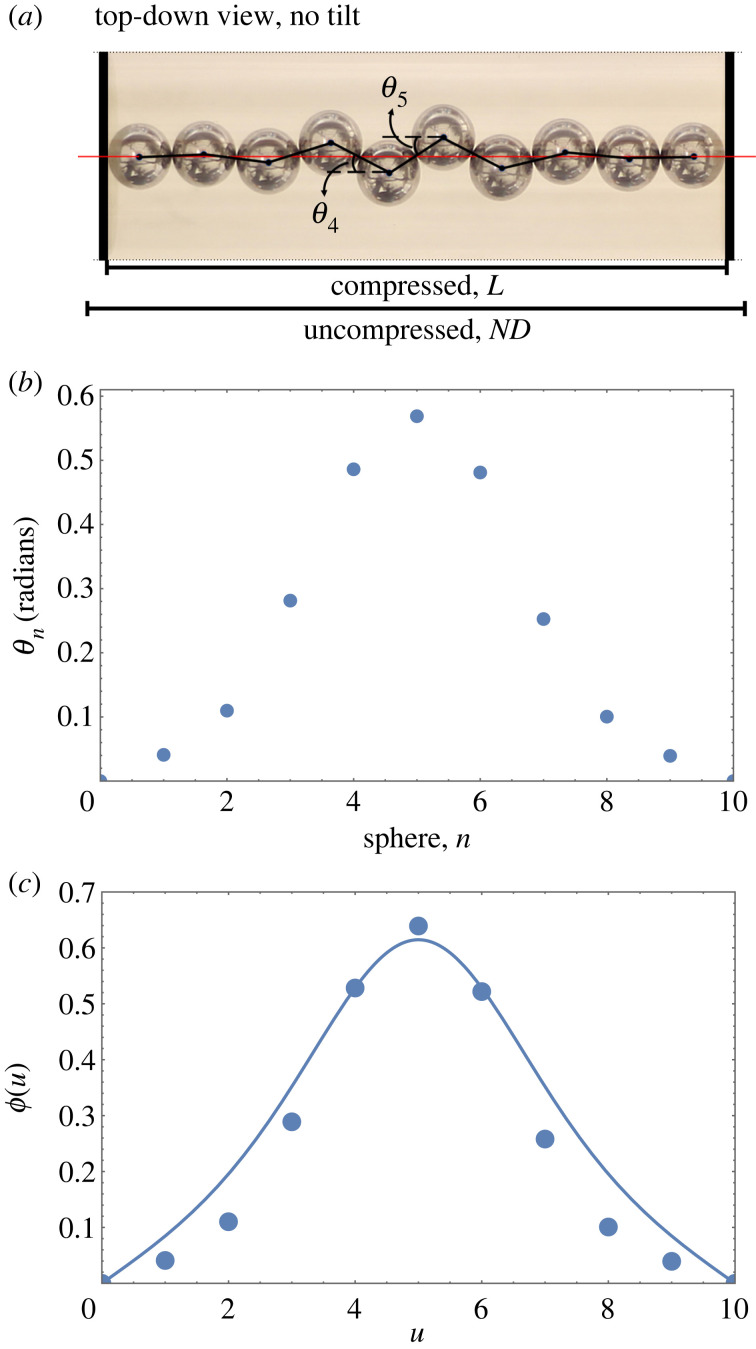
(*a*) Photograph of a buckled line of *N* = 10 metal spheres (ball bearings) resting in a horizontal cylinder, between two stoppers. In such an experiment, the curvature of the cylinder provides an approximately harmonic potential, that is, a restoring force acting on transversely displaced spheres [1,2]. The angles *θ*_*n*_ between contacting spheres and the cylinder axis vary along the line. (Inner cylinder diameter 21.05 mm, sphere diameter *D* = 6.44 mm, line length *L* = 61.6 mm, corresponding to a compression Δ = (*ND* − *L*)/*D* = 0.47 (see also equation (A7)).) Figure (*b*) shows the corresponding plot of the angle profile *θ*_*n*_. The continuum theory described in this paper yields a continuous profile for *ϕ*(*u*) = tan*θ*(*u*)). This is shown by the continuous line in (*c*), together with the experimental data from (*b*).

A line of hard spheres, confined in the transverse direction by a harmonic potential, buckles when compressed. In an experiment such a potential is (approximately) realized by confining the spheres in a horizontal cylinder [1,2]. As a sphere is laterally displaced, gravity provides an approximately harmonic restoring force due to the curvature of the cylinder. Figure 1*a* shows that upon compression of the line of spheres along the cylinder axis neighbouring spheres are displaced in opposite (alternating) directions. They form a zigzag profile whose amplitude is modulated and becomes increasingly localized with increasing compression. Figure 1*b* shows an example of a profile of the angle between contacting spheres, as defined in figure 1*a*.

Many stable and unstable equilibrium states exist, with localization in different places. A further dimension is added to the problem by the introduction of a longitudinal force on each sphere, for example, by tilting the system.

The discrete nature of the line of spheres is at the centre of the problem under consideration since the buckling of the line is solely due to sphere displacement. The spheres themselves do not deform; indeed, in the model which we present they are treated as infinitely hard. Buckling is a consequence of geometrical constraints.

The description of all this poses an interesting nonlinear problem, for which any predictions may be readily compared with experiments or contrasted with other phenomena involving the buckling of a linear chain of particles or rods under confinement. Examples of the latter include the transition of a linear chain of ions to a zigzag formation as observed in laser-cooled traps [3–9], dusty clusters [10,11], colloidal particles [12,13], droplets in microfluidic crystals [14], linear chains of magnetic spheres [15,16], as well as the classical problem of Euler buckling [17], or the buckling of thin rods confined inside (or on the surface) of a cylinder [18–21].

In earlier papers [1,2,22–25], we engaged in a numerical search for solutions, which were gathered up in detailed bifurcation diagrams (plots of energy, or another property, as a function of a control parameter, usually compression). This was done for up to *N* = 20 spheres [22]. The smooth modulation of the displacement profile that is generally found for large *N* (and small compression) invites the consideration of a continuous approximation, in terms of a profile that is a function of a continuous variable *u*, rather than an integer.

A continuous approximation was accordingly developed and applied to the case of a chain compressed between two movable hard walls, i.e. stoppers [23,25]. For low values of compression, it indeed worked well; for an example, see figure 1*c*. Moreover, it proved possible to reduce the differential equation at the heart of this description to a form that has Jacobi functions as exact solutions [25].

In the present paper, we return to the same theme, but with the inclusion of an additional longitudinal force acting on the spheres. Since this is readily realized in experiment by tilting the system, introducing a longitudinal component of gravity, we often refer to it as ‘tilt’.

**Figure 2.**
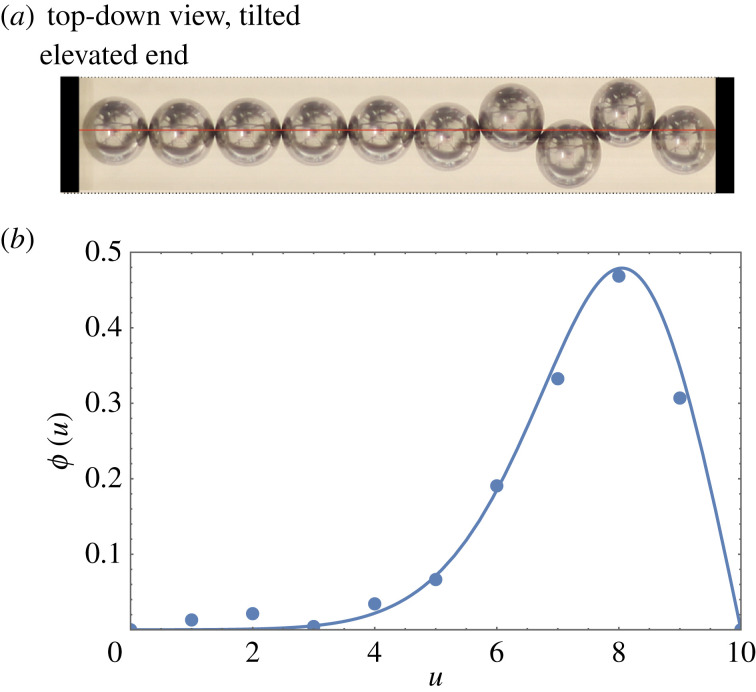
(*a*) View of a compressed and tilted linear line of spheres; the maximum of sphere displacement has moved towards the lower end of the cylinder. (Cylinder and sphere dimensions as in figure 1, chain length 61.56 mm, tilt angle $\alpha ≃7^{\circ }$.) (*b*) Theoretical results from the continuum model (§2), compared with the measured profile, in terms of *ϕ*(*u*) = tan*θ*(*u*) (cf. figure 1).

Previously, we have addressed the effect of tilt where the upper end of the chain is not subject to a compressive force [2]. In what follows we study the general case, including finite compressive force, using a continuous theory. The typical form of these results is illustrated by figure 2, to be compared with figure 1. Remarkably, our analysis leads to further formulations in terms of special functions—the Whittaker and Airy functions. Hence a transparent description of a wide range of situations, in which tilt and compression may both be implicated, is possible. To this claim must be added the reservation that when large amplitudes of buckling are reached, the description is necessarily inadequate, except for qualitative purposes.

The structure of the present article is a follows. In §2, we introduce the nonlinear differential equation of the continuum model, equation (2.2), and show some sample solutions for specific values of compression and tilt. In §3, we present several approximations to the equation which allow for *analytical* solutions in terms of scaled Jacobi, Airy, and Whittaker functions, respectively. The properties of these solutions are discussed in §4, examples are shown in figure 4. In §5, we present our results in the form of a phase diagram; a brief outlook is given in §6. Mathematical details are mostly confined to five appendices.

The progression of successive approximations in our mathematical description is outlined in table 1, as a guide to the sections that follow, and relevant publications. All experimental and numerical data shown in the following is for *N* = 10 spheres (data for higher *N* contains the same key features [1,22]); in our analytical expressions *N* enters simply as a parameter.

**Table 1.** Outline of the different scenarios, successive approximations and differential equations discussed in the text (see in particular §3), together with some relevant references. Examples of profiles for Jacobi, Airy and Whittaker solutions are shown in figure 4.

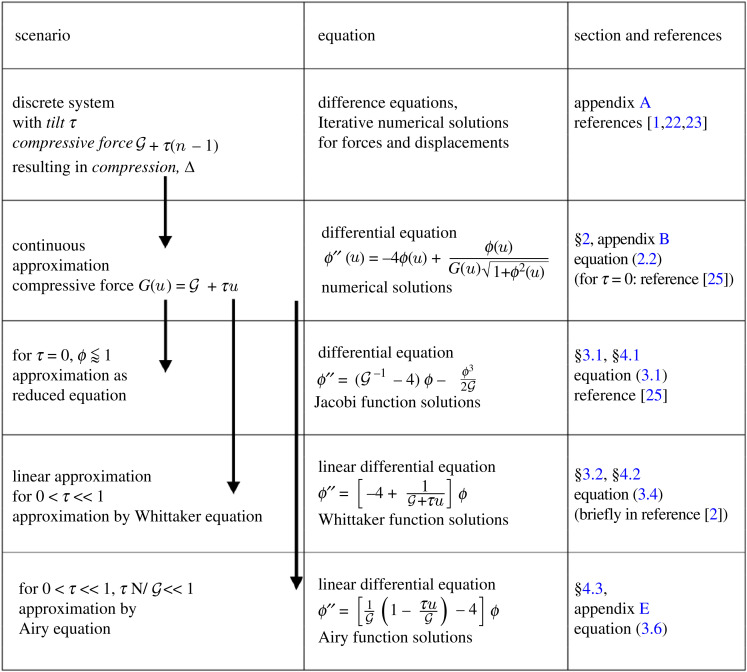

## 2. The continuous formulation

### 2.1. Differential equation

Equilibrium configurations of contacting hard spheres in a harmonic confining potential (with tilt), as shown in figures 1 and 2, result from a balance of forces. In appendix A, we show how the force balance leads to a set of iterative equations for the transverse forces acting on the spheres, and the angles *θ*_*n*_ (see figure 1*a* for notation) between successive lines of contact and the longitudinal direction. These discrete equations have been solved numerically using the shooting method [1,22–25], and the solutions produce the angle profiles, *θ*_*n*_.

In the absence of tilt, the discrete equations were also reformulated as a second-order differential equation [2,23,25] for which it is convenient to use as dependent variable *ϕ*(*u*), where

2.1 ϕ(u)=tan⁡θ(u).

The variable *u* replaces the index *n* in the discrete formulation. In appendix B, we derive the differential equation in the presence of tilt as

2.2 $$\phi^{\prime \prime }(u)=-4\phi (u)+\frac{\phi (u)}{G(u)\sqrt{1+\phi^{2}(u)}};$$

see also equation (B 5). In this ‘full continuum equation with tilt’, the axial component of the (dimensionless) compressive force *G*(*u*) varies linearly with position *u*, G(u)=G+τu (see also appendix A, figure 12). Here G is the magnitude of the compressive force (in the axial direction) at the (possibly elevated) end (*u* = 0) of the chain of *N* spheres and *τ* is the dimensionless *tilt* parameter. (In the experiments, *τ* is proportional to the sine of the angle of tilt; see equation (A1).) We note that for the case considered here there are only repulsive forces, so *G*(*u*), and in particular G is never negative.

Equation (2.2) is the foundation of this paper. We will examine its solutions for the hard-wall boundary conditions *ϕ*(0) = *ϕ*(*N*) = 0 (which corresponds to hard walls that are perpendicular to the cylindrical axis, see figure 1) and given values for compressive force at the upper (elevated) end, G, and tilt *τ*.

Equation (2.2) was previously only presented and analysed in this form for the case *τ* = 0, where Jacobi functions provide approximate solutions [25]. The presence of tilt (i.e. a finite value for *τ*) leads to further (approximate) analytical solutions, now in terms of Airy and Whittaker functions. (One displacement profile involving a Whittaker function was shown already in [2], but all mathematical background was omitted at the time.)

### 2.2. Compression

In contrast to our experimental set-up, where we fix compression by choosing the distance between the two stoppers at the ends of the chain, here we *compute* profiles for given tilt *τ* and various values G, evaluating compression Δ from the profile *ϕ*(*u*) via

2.3 $$\Delta =N-\int_{0}^{N}\frac{du}{\sqrt{1+\phi^{2}(u)}};$$

see also appendix C, equation (C1).

As with experimental data, we can thus plot quantities of interest such as peak position or peak height as a function of compression.

### 2.3. Numerical results

Before proceeding to analytic approximations, it is instructive to briefly show numerical results for solutions of the full equation, equation (2.2). The equation is solved using for example Mathematica, with the required boundary conditions *ϕ*(0) = *ϕ*(*N*) = 0.

#### 2.3.1. Numerical results for compression only

**Figure 3.**
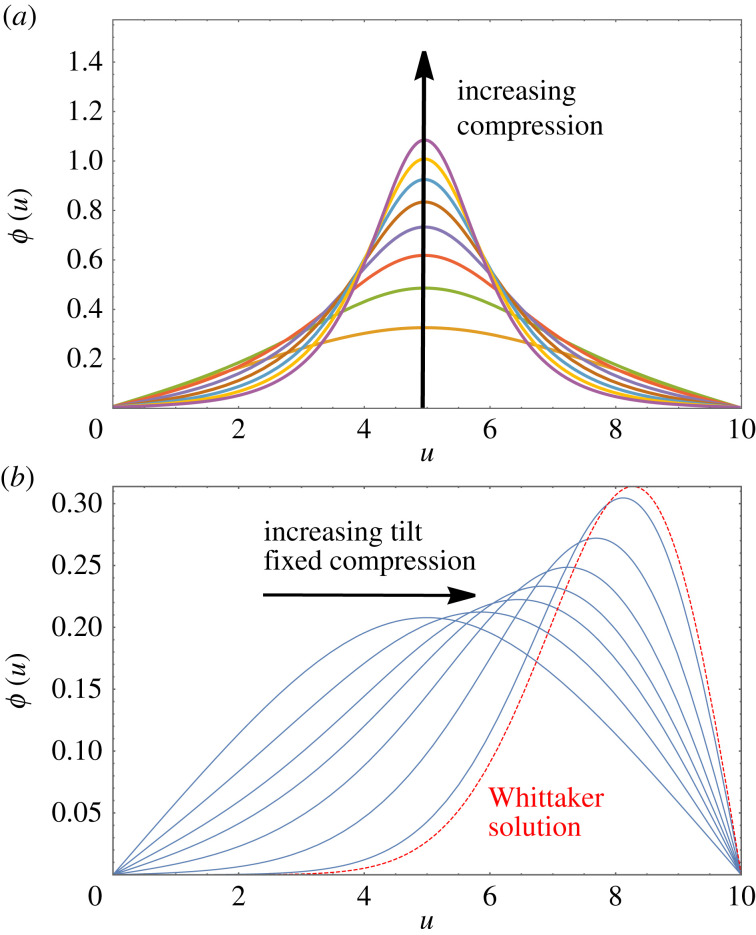
Results from numerical solutions of the full equation, equation (2.2), for a chain of *N* = 10 spheres under compression. (*a*) Examples of profiles of *ϕ*(*u*) in the absence of tilt (*τ* = 0) for compressive forces in the range G=[0.185,0.257], resulting in compression Δ in the range from 0.1 to 0.6. (*b*) For fixed compression (here Δ = 0.1), the introduction of a finite value of tilt leads to a shift of the peak away from the centre. Shown as a red dashed line is the limiting case for the absence of a compressive force at the elevated end (*u* = 0), i.e. G=0, at Δ = 0; this is the Whittaker solution, equation (4.2), which we discuss in §4.2.

Figure 3*a* shows examples of profiles of *ϕ*(*u*) for *N* = 10 in the absence of tilt (*τ* = 0), computed for several values of the compressive force G. The profiles are symmetric around the centre of the chain.

In the limit in which compression Δ → 0, the peak height varies as *ϕ*_max_ ∼ Δ^1/2^, with a linear variation for higher values of compression [23]. The square-root scaling for small compression is obtained by approximating *ϕ*(*u*) as a triangular profile of peak height *ϕ*_max_ and using $\Delta ≃(1/2)\int_{0}^{N}\phi^{2}(u)\;du$ (see equation (C 2)). We will return to this in §4.1 when discussing analytical solutions of a reduced equation in terms of scaled Jacobi functions.

#### 2.3.2. Numerical results for compression and tilt

In figure 3*b*, we show that the presence of a finite tilt results in a shift of the peak away from the centre of the chain (as seen in figure 2). A detailed discussion of this case will be provided in §4.

## 3. Approximations of the full equation

**Figure 4.**
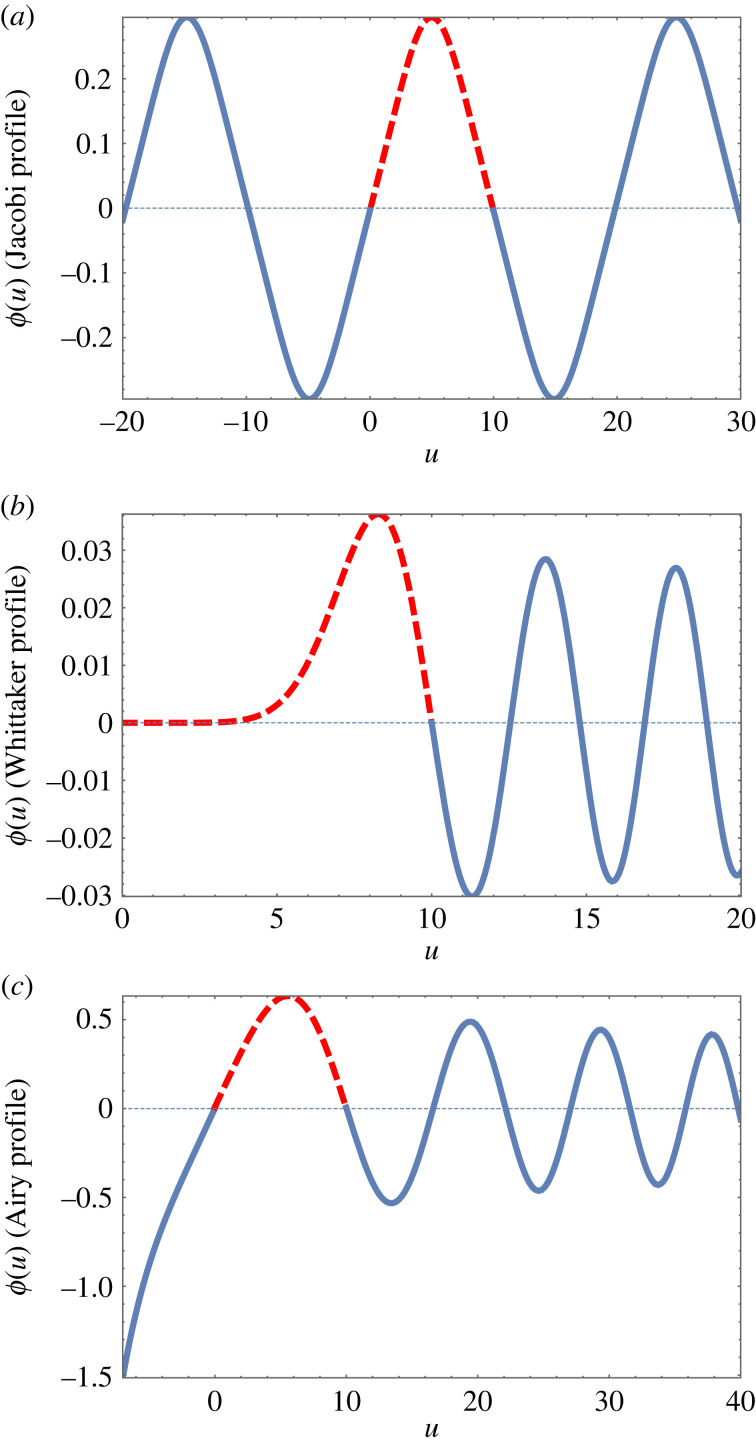
Examples of the analytic functions used in this work, shown over a wide range. Only sections such as those displayed as red-dashed lines are of relevance here; they fulfil the required boundary conditions, *ϕ*(0) = 0 and *ϕ*(*N* = 10) = 0.

The differential equation, equation (2.2), may be reduced by various reasonable approximations, resulting in forms which have analytic solutions, as summarized in table 1 and shown in figure 4. These provide insight into the properties of (numerical) solutions of equation (2.2) for different ranges of compression and tilt (§5).

### 3.1. The case of small compression in the absence of tilt

We first consider the absence of tilt, *τ* = 0, for which there is a constant compressive force G(u)=G. Taylor expanding the square root in the denominator of the right-hand side of equation (2.2) to order *ϕ*^2^, one obtains the *reduced* equation [25],

3.1 $$\phi^{\prime \prime }=(G^{-1}-4)\phi -\frac{\phi^{3}}{2G}.$$

(In [25] this was written in terms of a parameter *κ*^2^, defined as $\kappa^{2}=G^{-1}-4$.)

By making an appropriate change of variables, $\phi =2\sqrt{\left(m/(2m-1))(1-4G\right)}y$, and $u=\sqrt{(2m-1)(G/(1-4G}))x$ this can be re-written in the form of the *Jacobi differential equation*

3.2 $$y^{\prime \prime }=-(1-2m)y-2my^{3},$$

with 0 < *m* < 1. Its analytical solution *y*(*x*) = *cn*(*x*|*m*) is the Jacobi *cn* function [26,27], $\sqrt{m}$ is called the (elliptic) modulus. A detailed discussion of the properties of the *scaled* Jacobi *cn* functions, which are the solutions of equation (3.1), is given in [25]; see also the brief discussion in §4.1 and appendix D.

### 3.2. Compression and tilt

In the case of finite tilt, *τ* > 0, analytical solutions in terms of Whittaker or Airy functions are available if one neglects the *ϕ*^2^ term in the denominator of the square root in the full equation, equation (2.2), and thus considers the *linear* equation

3.3 $$\phi^{\prime \prime }=\left[-4+\frac{1}{G+\tau u}\right]\phi .$$

#### 3.2.1. Whittaker equation

By introducing $\tilde{u}=u+G/\tau$, we put this in the form of

3.4 $$\phi^{\prime \prime }=\left[-4+\frac{1}{\tau \tilde{u}}\right]\phi .$$

Equation (3.4) is a special case of the *Whittaker Equation* [28]

3.5 $$\frac{d^{2}w}{dz^{2}}=\left[\frac{1}{4}-\frac{k}{z}+\frac{\left((1/4)-\mu^{2}\right)}{z^{2}}\right]w,$$

for $z=4i\tilde{u}$, *μ* = 1/2, *k* = 1/(4*τ*), and renaming *w* as *ϕ*.

In this paper, we concentrate on the case G=0 (i.e. $\tilde{u}=u$), corresponding to experiments in which the upper end of the chain is not in contact with the wall. The exact solutions of equation (3.4) for our boundary conditions can then be written in terms of Whittaker functions, *M*_*k*,*μ*_(*z*) [28]; see §4.2.

The Whittaker solution defines only a single solution for a given value of *N,* resulting in a prediction for the critical value of tilt, where G=0, for that given *N*. In order to explore the effect of tilt *and* compression on the system, we make a different approximation which leads to the Airy equation, as discussed in the next section.

#### 3.2.2. Small tilt and a finite compressive force: Airy equation

For finite G and τN/G≪1, we may retain only the lowest order term in τu/G in equation (3.3), resulting in:

3.6 $$\phi^{\prime \prime }=\left[\frac{1}{G}\left(1-\frac{\tau u}{G}\right)-4\right]\phi .$$

By making a change of variable from *u* to an appropriately defined *x* (see appendix E) and renaming *ϕ* as *y* we obtain the familiar *Airy equation*,

3.7 $$y^{\prime \prime }(x)=xy(x),$$

which has analytical solutions in terms of the Airy *Ai* and *Bi* functions. In appendix E we derive the exact solutions of equation (3.6) in terms of these Airy functions; we will discuss the properties of these solutions in §4.3.

### 3.3. The form of the analytical solutions

Figure 4 shows examples of the special functions used here, over an extended range. One may choose as a solution (with hard wall boundary conditions) any range between two zeroes (corresponding to the boundary conditions Φ=0 at each end). However, those that have internal zeroes have higher energies and are not considered here.

In the next section, we explore the properties of the analytic solutions in terms of Jacobi, Airy and Whittaker functions.

## 4. Properties of analytical solutions

Analytical solutions result in relationships between the experimental parameters compression, tilt and the number of spheres, and the observed quantities peak position and peak height. They also enable us to predict the critical value of tilt for sphere detachment (where G(0)=G=0).

Each of the analytical solutions offers a different, limited perspective on experimental features. Using Jacobi functions, one arrives at the relation between peak height and compression in terms of elliptic integrals. Using Airy functions establishes a relation between the position of the profile maximum and tilt. Whittaker functions enable a prediction of the critical value of tilt at which detachment of the spheres from the top wall occurs for (low values of) fixed compression.

### 4.1. Compression only: Jacobi functions

For values of compression Δ≲0.3, and in the absence of a longitudinal tilt force, solutions of the full continuum equation are well approximated by scaled Jacobi functions. An example of such a profile was already shown in figure 4*a*.

For the case of the hard wall boundary conditions considered here, i.e. *ϕ*(0) = *ϕ*(*N*) = 0, the solution of the reduced equation in the absence of tilt (*τ* = 0), equation (3.1), is given in terms of the scaled Jacobi *cn* function as

4.1 $$\phi (u)=\phi_{\max}cn\left(\sqrt{\frac{\left(G^{-1}-4\right)}{\left(2m-1\right)}}(u-N/2)|m\right),$$

where the so-called modulus, $\sqrt{m}$, is related to the period of the Jacobi functions, and thus *N*. The derivation of this solution, and a discussion of its properties, is found in our recent publication [25], with further details in appendix D. (In [25], relevant equations and quantities are expressed in terms of $\kappa^{2}=G^{-1}-4$ instead of G.)

**Figure 5.**
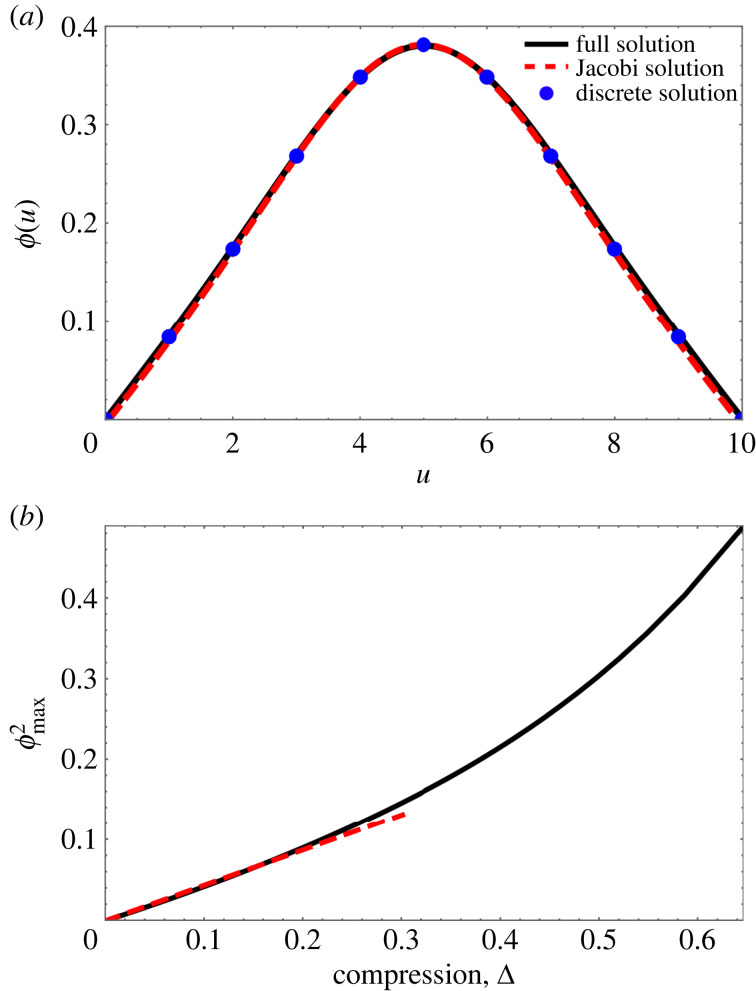
In the absence of tilt and for small compression, scaled Jacobi functions provide good approximate solutions of the full continuous differential equation, equation (2.2). (*a*) Profile *ϕ*(*u*) for the case of *N* = 10 with a compressive force G=0.2435. The solid black line is a numerical solution for *ϕ*(*u*) for equation (2.2). The dashed red line is the analytical solution of the reduced equation, equation (3.1), in terms of a scaled Jacobi function. Also shown are the data points for the corresponding discrete system (appendix A). The computed values of compression Δ for the three profiles are Δ_full_ = 0.30, Δ_discrete_ = 0.30, Δ_Jacobi_ = 0.28. (*b*) The peak height *ϕ*_max_ increases with compression. The dashed red line, $\phi_{\max}^{2}=0.43\Delta$, shows an analytical result for small compression (equation (D 5)).

Figure 5*a* shows the profile *ϕ*(*u*) as obtained from discrete simulations, a numerical solution of the full continuum equation, equation (2.2), and the Jacobi solution, equation (4.1) for Δ ≃ 0.3. For small values of compression, the maximum value of *ϕ* increases as $\phi_{\max}\propto \sqrt{\Delta }$; see figure 5*b* and appendix D, equation (D 5).

### 4.2. Whittaker functions and the critical tilt for detachment

From experiments and numerical solutions for both the discrete system and the full continuum equation (figure 3*b*), one finds that (for given compression Δ) there is a critical value of tilt, *τ*_*c*_(Δ), beyond which the spheres detach from the upper boundary, so that the compressive force there goes to zero, i.e. G(u=0)=G=0.

**Figure 6.**
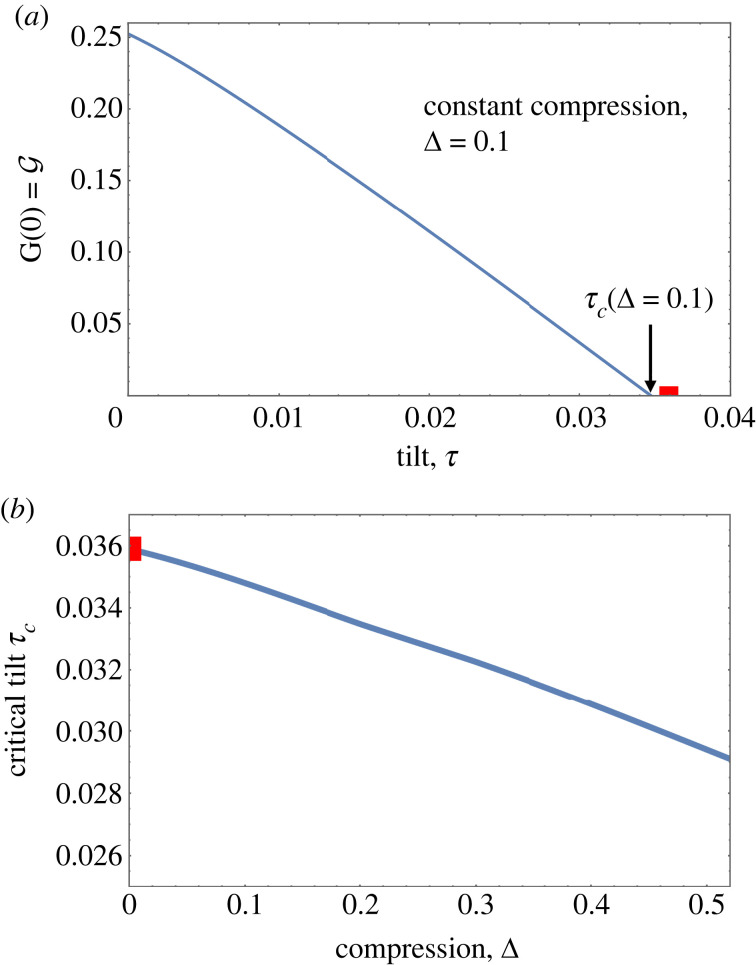
(*a*) Variation with tilt of the compressive force G=G(u=0) at the elevated ‘top end’ of the chain, for compression Δ = 0.1. Detachment of the chain corresponds to G=0. (*b*) The variation of the critical value of tilt (where G=0) as a function of compression for the full numerical solutions of the continuum equation with *N* = 10. The red square at Δ = 0 corresponds to the prediction made from the Whittaker solution.

As an example we show in figure 6*a* the decrease of G(0)=G with tilt for constant compression Δ = 0.10, as obtained from numerical solutions of the full equation, equation (2.2) for *N* = 10. The critical tilt for detachment is determined as *τ*_*c*_(0.1) = 0.0345. Its variation with compression is shown in figure 6*b*; we will show below how the value of the critical tilt for the uncompressed system, Δ = 0, is determined analytically using Whittaker functions.

We saw in §3.2.1 that for small *ϕ* the full equation can be approximated by a special case of the Whittaker equation, equation (3.4). For G=0 and the boundary condition *ϕ*(*u* = 0) = 0 and a given value of *ϕ*′(0) (which is chosen so to obtain a specified compression) its solution is given by

4.2 $$\phi (u)=\frac{\phi^{\prime }(0)}{4i}M_{\left(i/4\tau ),(1/2\right)}(4iu),$$

where *M* is the Whittaker function [28] and *i* is the unit imaginary number. (Here we have used the property of the Whittaker function, (d/d*u*)*M*_(*i*/4*τ*),(1/2)_(4*iu*)|_*u*=0_ = 4*i*). An example of this solution was shown in figure 4*b*.

The chosen value of tilt *τ* in equation (4.2) uniquely determines the distance between the zero of *ϕ*(*u*) at *u* = 0 and its first zero at a positive value of *u*. Rephrased in the context of this manuscript: for a given number *N* of spheres (requiring *ϕ*(*N*) = 0 for our boundary conditions) there is a well-defined critical value of tilt, *τ*_*c*_, for detachment (i.e. G(u=0)=G=0 at *τ*_*c*_). This value can be determined (numerically) from equation (4.2); an upper bound estimate is given by *τ*_*c*_ < [3(*N* − 2)]^−1^ [2].

For the case *N* = 10, the boundary conditions *ϕ*(0) = *ϕ*(10) = 0 are fulfilled for *τ*_*c*_ = 0.0359. From figure 6*b*, we see that this is the critical value of tilt for detachment at compression Δ = 0, i.e. *τ*_*c*_(Δ = 0) = 0.0359. For higher values of compression, the value of tilt *τ*_*c*_ is reduced; see figure 6.

For values of tilt close to detachment, and for small values of compression, the Whittaker solution serves as an analytic approximation of the numerical solutions of the full equation. Figure 3*b* demonstrates this for the case of compression Δ = 0.1.

**Figure 7.**
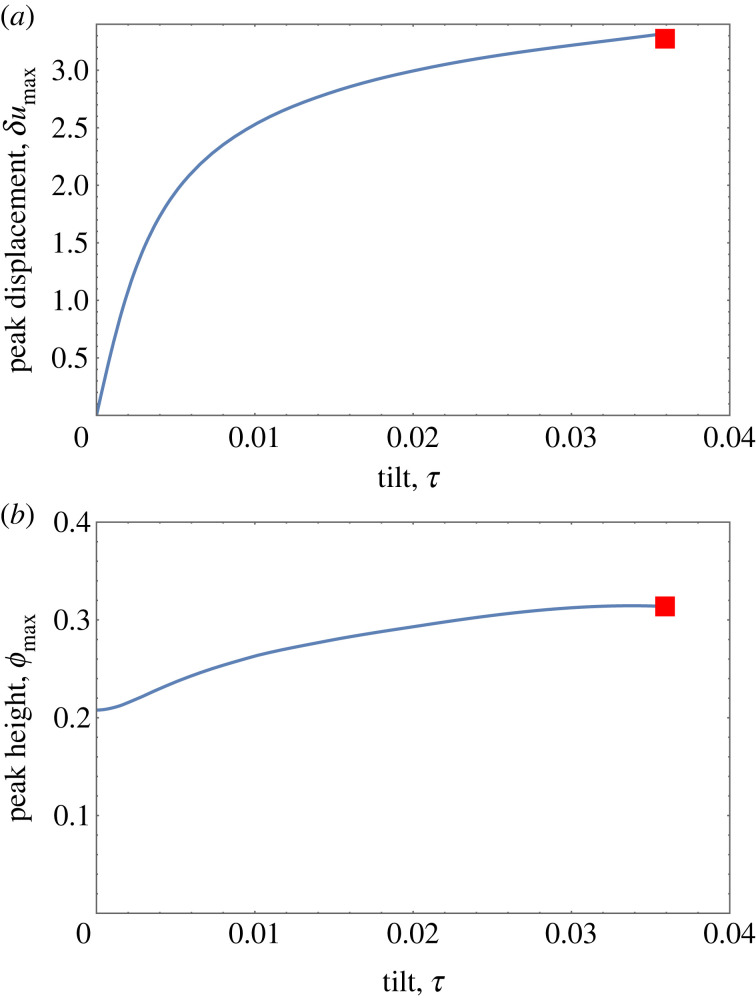
Results from numerical solutions of the full equation, equation (2.2), for the tilting of a chain under constant compression (Δ = 0.10). (*a*) The introduction of tilt leads to a displacement, *δu*_max_, of the peak position away from the centre. (For *N* = 10 as shown here: *δu*_max_ = *u*_max_ − 5.) (*b*) Variation of peak height with tilt. In each case, the corresponding result of Whittaker theory (for Δ = 0) is shown by a red square.

The Whittaker solution also provides an estimate for both peak position and peak height at detachment. Figure 7*a* shows numerical results for the displacement of the peak away from the centre as a function of tilt, at fixed compression Δ = 0.1, as obtained from numerical solutions of the full equation, equation (2.2). For small values of *τ*, this is linear in *τ*. At detachment, the peak remains a finite distance away from the bottom wall. Shown as a red square in figure 7*a* is the peak displacement of the Whittaker solution, equation (4.2), corresponding to detachment at Δ = 0.

**Figure 8.**
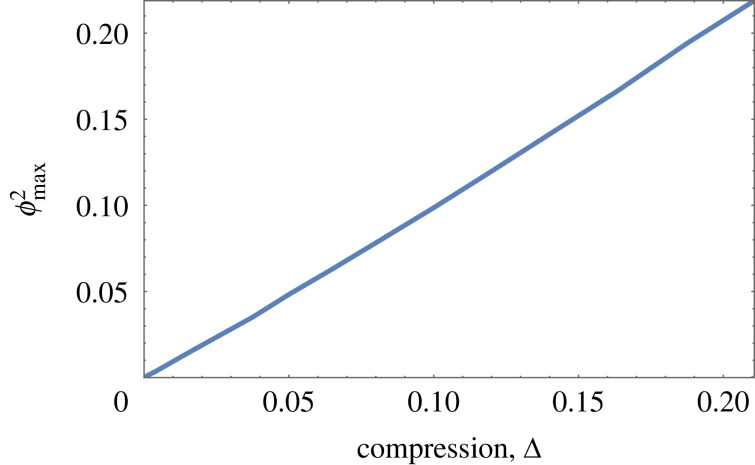
The peak height *ϕ*_max_ of the scaled Whittaker solutions, equation (4.2), varies approximately as $\phi_{\max}^{2}≃\Delta$. For small compression, Δ, this approximates the behaviour of the full solution near detachment.

The variation of peak height, *ϕ*_max_, as a function of tilt for fixed compression is shown in figure 7*b*. This too converges to a finite value at the point of detachment, which is well approximated by the Whittaker solution for this compression. The peak height of a Whittaker profile scales as the square root of compression Δ, as shown in figure 8.

### 4.3. Compression and tilt: Airy functions

The Airy function formulation reproduces some of the results from Whittaker functions, at least qualitatively, in a much more familiar form. It provides the following approximate solution of the full equation

4.3 $$\phi (x)=c_{1}Ai(x)+c_{2}Bi(x).$$

Here the variable *x* is given by

4.4 $$x={\left(\frac{\tau }{G^{2}}\right)}^{-2/3}\left[\frac{1}{G}\left(1-\frac{\tau u}{G}\right)-4\right],$$

and the constants *c*_1_ and *c*_2_ are determined from the boundary condition *ϕ*(0) = *ϕ*(*N*) = 0, see appendix E.

The validity of the Airy solution is restricted by the validity of the condition Nτ/G≪1 (table 1); all numerical results in this section conform to that condition.

**Figure 9.**
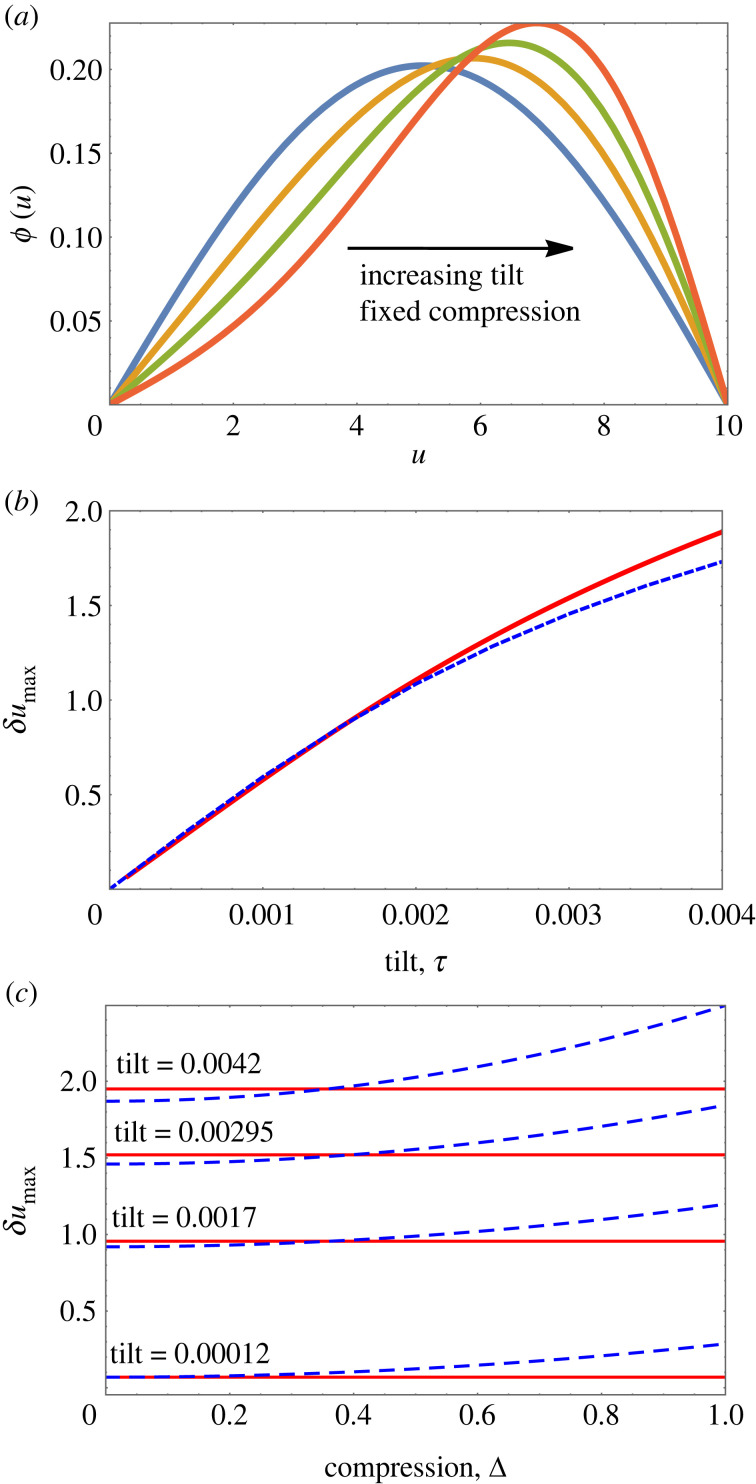
For small values of both tilt *τ*, and compression Δ, combinations of Airy *Ai* and *Bi* functions reproduce the key feature of solutions of the full continuum equation, namely displacement of the profile peak away from the centre, in response to the application of tilt. (*a*) Profiles for *ϕ*(*u*) in terms of Airy functions for fixed compression Δ = 0.1 and varying tilt *τ* in the range (0, 0.0045) (for *N* = 10). (*b*) Peak displacement *δu*_max_ (as in figure 7*a*), as a function of tilt for Δ = 0.1. Blue dashed line: result from the full equation, equation (2.2). Red solid line: Analytical result involving Airy functions. (*c*) Peak displacement as a function of compression for several values of tilt. Blue dashed line from full equation, red solid line: Airy solution.

Figure 9*a* shows examples of profiles *ϕ*(*u*), obtained for fixed values of compression, and several values of tilt. For small values of tilt *τ*, the position of the maximum varies linearly with *τ* (figure 9*b*). (The linear variation for small *τ* was already seen in the numerical solutions of the full equation; see figure 7*a*.)

Equation (3.6) (of which equation (4.3) is an exact solution) is linear in *ϕ*, allowing for a simple scaling of its solutions to obtain profiles corresponding to different values of compression, Δ. The peak position is thus only dependent on tilt, but independent of compression. This is shown in figure 9*c* where we contrast this behaviour with that of solutions of the full equation.

## 5. Phase diagram and energy

**Figure 10.**
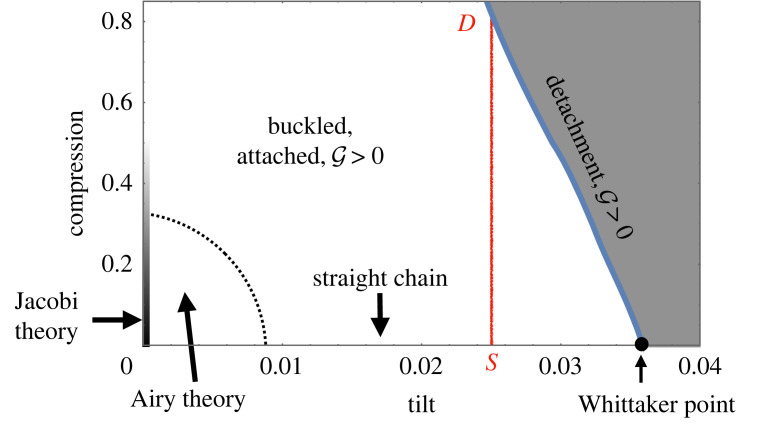
Phase diagram representing the results of the continuum model (for *N* = 10) in the plane of tilt *τ* and compression Δ. The solid blue line demarcates a phase boundary and is defined by G(0)=G=0, i.e. detachment at the elevated end of the chain of spheres. The area to the left of it corresponds to buckled structures, the area to its right to collapsed structures. In the case of compression Δ = 0 (horizontal axis), the chain remains unbuckled up to the value of critical tilt *τ*(Δ = 0) = 0.0359 which is given by the Whittaker solution. Knowledge of G(Δ) along a vertical line enables the calculation of the energy difference between any two states using the work energy theorem (for example, between the points *S* and *D* indicated on the diagram).

The results of our investigation can be presented in the form of a phase diagram with axes tilt *τ* and compression Δ. The phases that we identified are the straight chain (*τ*_*c*_(Δ = 0)), the buckled attached chain (G>0), and detached states. All these are marked up in figure 10 for the case *N* = 10, together with an indication of the validity ranges of the various analytical solutions for the profiles *ϕ*(*u*).

Also indicated in the phase diagram is a straight line (corresponding to constant tilt, *τ* = 0.025) leading from the point *S* (unbuckled tilted chain) to *D* (buckled chain, at the point of detachment). Knowledge of the compressive force G(Δ) at the elevated end of the chain as a function of compression, Δ, allows for the computation of the energy difference between two states along that line using the work energy theorem

5.1 $$E-E_{S}=\int_{0}^{\Delta }G(\Delta )\;d\Delta .$$

**Figure 11.**
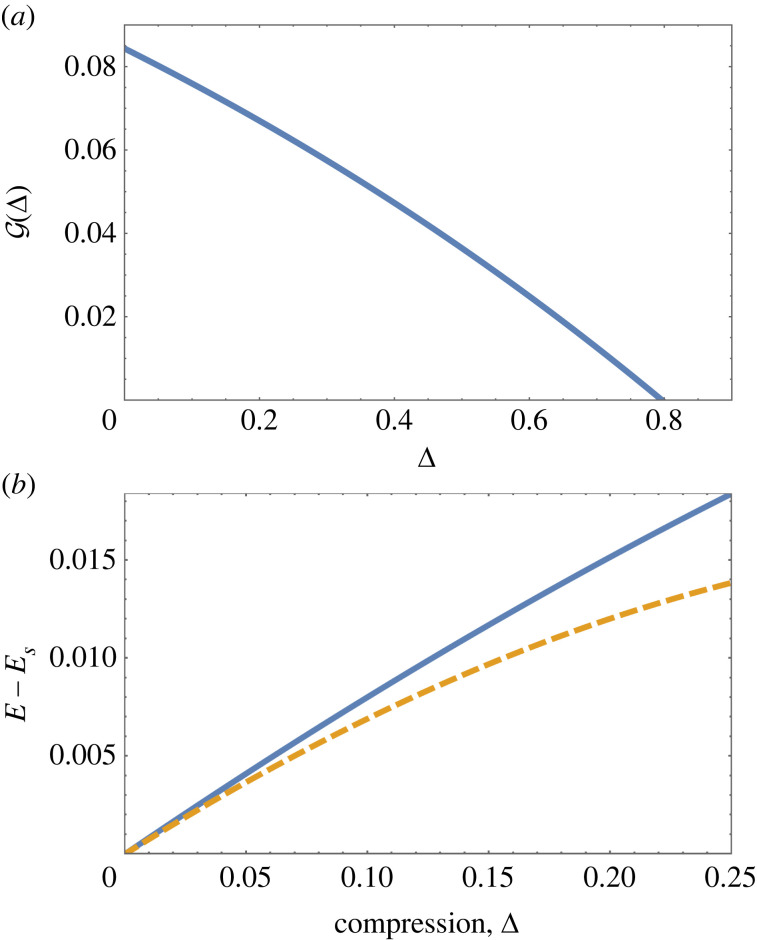
(*a*) Variation of G with compression Δ, for constant tilt *τ* = 0.025 (corresponding to the vertical red line in the phase diagram, figure 10). Detachment occurs at the intersection with the horizontal axis, at Δ = 0.795. (*b*) Energy, relative to the energy of a tilted straight chain, as function of compression for fixed tilt *τ* = 0.025. Solid blue line: work-energy theorem, equation (5.1). For low values of *ϕ*, the energy can also be expressed in the integral form of equation (5.2), see dashed yellow line.

Figure 11*a* shows that G decreases with compression and vanishes at Δ ≃ 0.795 (for tilt *τ* = 0.025), corresponding to detachment. Numerical integration of G(Δ) using the work-energy theorem, equation (5.1) results in the energy difference *E* − *E*_*S*_, as shown in figure 11*b*.

**Figure 12.**
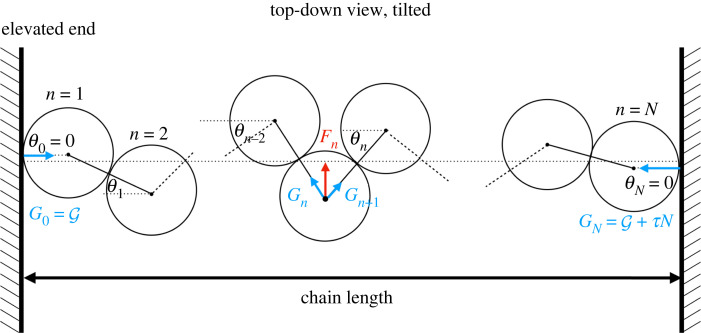
Schematic diagram for a chain of hard spheres under compression. In non-dimensional units, the transverse restoring forces *F*_*n*_ equal the transverse displacements. *G*_*n*_ is the compressive force between contacting spheres *n* − 1 and *n*. G is the compressive force exerted by the top wall on the first sphere, *n* = 1. The angles *θ*_*n*_ are defined so as to be always positive for the modulated zig-zag structures discussed here (hard walls: *θ*_0_ = *θ*_*N*_ = 0).

An alternative route to evaluate this energy difference is as follows. The total energy of a line of spheres has two contributions. Tilting the line away from the horizontal leads to a gravitational energy; it is given by *E*_*S*_ = *τN*^2^/2, if the chain is straight (see equation (A 9)). Buckling, i.e. sphere displacement in the transverse direction, results in a second contribution, associated with the harmonic confining potential. The resulting expression for the total energy, equation (A 8), is derived in appendix A.

An equivalent expression for total energy in the continuous formulation is shown in appendix B (equation (C 4)),

5.2 $$E≃\frac{1}{8}\int_{0}^{N}\frac{\phi^{2}(u)}{1+\phi^{2}(u)}\;du+\tau \left(\int_{0}^{N}\frac{u\;du}{\sqrt{1+\phi^{2}(u)}}\right)≃\frac{\Delta }{4}-\frac{\tau }{2}\int_{0}^{N}u\phi^{2}(u)\;du.$$

This equation allows for the direct computation of the energy from solutions *ϕ*(*u*) of the full continuum equation, equation (2.2). Figure 11*b* shows that this expression replicates the linear increase in energy for low compression, i.e. solutions with small values of *ϕ*.

## 6. Outlook

The analysis presented here was stimulated by some simple experiments [1,2,22] and should in turn provide more systematic investigations. In addition, the generalization of the buckling problem to the case of elastically deformable spheres (of which bubbles are an obvious case, or hydrogel spheres) has only been touched upon [1,29]. While it is unlikely to be amenable to the kind of analytic treatment given here for hard spheres, it must relate to it as a limiting case. Experimentation with bubbles would also eliminate friction, which may stabilize otherwise unstable structures [2], but is not included in the theory described here.

A major limitation of the present work is its breakdown at large buckling amplitude, which entails transitions to states that we have termed ‘doublets’ [23], where second-neighbour contacts arise. One may then envisage a phase diagram of the type shown in figure 10 which in addition to the straight and buckled phases also includes the doublet states, and, for soft spheres, a compressed straight phase. We intend to determine the corresponding phase boundaries in due course.

Finally, all the solutions presented in this paper are lowest energy solutions, corresponding to the ‘ground state’ of the buckled chain. Higher energy solutions exist [23], but were not considered here.

## Data Availability

The computations given in the paper were made using Mathematica. Use the following link to download the Mathematica file for the relevant computations: https://www.dropbox.com/s/i2uhvxh21ye0u29/continuumDescriptionOfBucklingSpheresMATHEMATICA.nb?dl=0. This file can be run using Mathematica 13 to reproduce our calculations. For those without access to Mathematica, we provide a pdf print out of the Mathematica file: https://www.dropbox.com/s/bc38p4v9g5hwlfq/continuumDescriptionOfBucklingSpheresMATHEMATICA.pdf?dl=0. The data are provided in electronic supplementary material [30].

## Appendix A. Modelling the discrete system

In our model, a line of *N* contacting identical hard spheres is compressed between hard walls, as in figure 12. Displacement of a sphere by a distance *R*_*n*_ away from the central axis results in a transverse restoring force *f*_*n*_ with magnitude *k R*_*n*_, where *k* is a force constant.

In *experiments* with hard spheres, placed at the bottom of a horizontal cylinder, the restoring transverse force is provided by gravity, as a transversely displaced sphere experiences the curvature of the cylinder. It can be shown that for the magnitudes of transverse displacements encountered in such a set-up, the restoring force is approximately linear in this displacement, with a force constant *k* given by *k* = 2 *mg*/(*d* − *D*) [2]. Here *m* and *D* are, respectively, sphere mass and diameter, *d* is the cylinder diameter, and *g* is acceleration due to gravity.

We introduce non-dimensional quantities by defining *r*_*n*_ = *R*_*n*_/*D*, where *D* is the sphere diameter. The dimensionless transverse force *F*_*n*_ is defined as *F*_*n*_ = *f*_*n*_/(*kD*). Inserting for *f*_*n*_ leads to *F*_*n*_ = *r*_*n*_; in our non-dimensional formulation the transverse force *F*_*n*_ acting on a sphere equals its transverse displacement *r*_*n*_, a positive quantity.

In a line of spheres which is tilted by an angle *α* with respect to the horizontal, each sphere of mass *m* experiences a tilt force *mg*sin*α* along the axial direction. In the following, we introduce the non-dimensional tilt variable *τ* by

A 1 $$\tau =\frac{mg\sin\alpha }{\left(kD\right)}.$$

The axial component of the compressive force between contacting spheres depends on the position of a sphere within the line, according to,

A 2 $$G_{n}\cos\theta_{n-1}=G+\tau (n-1),$$

from the condition of force equilibrium. G is the magnitude of the compressive force exerted by the wall on the sphere, *n* = 1 at the (possibly) elevated end of the line.

Transverse force balance for the displaced *n*th sphere is expressed as

A 3 $$F_{n}=G_{n}\sin\theta_{n-1}+G_{n+1}\sin\theta_{n}=(G+\tau (n-1))\tan\theta_{n-1}+(G+\tau n)\tan\theta_{n},$$

and we thus obtain

A 4 $$\tan\theta_{n}=\frac{F_{n}-(G+\tau (n-1))\tan\theta_{n-1}}{G+\tau n}.$$

The centres of contacting spheres are separated by their diameter. Hence in our dimensionless variables the radial distances and forces are given by

A 5 $$F_{n}+F_{n+1}=r_{n}+r_{n+1}=\sin\theta_{n}.$$

Rewritten in terms of *ϕ*_*n*_ = tan*θ*_*n*_, the two iterative equations are thus

A 6 $$\begin{array}{cc} & \phi_{n}=\frac{F_{n}-(G+\tau (n-1))\phi_{n-1}}{G+\tau n}\\ \mathrm{and}\;\;\;\;\;\;\; & F_{n+1}=\frac{\phi_{n}}{\sqrt{1+\phi_{n}^{2}}}-F_{n}, \end{array}\}$$

These equations can be used in a shooting method to find solutions for a specified value for G [22,23]. The hard wall boundary condition for sphere *n* = 1 requires the first angle *θ*_0_ to be zero (thus also *ϕ*_0_ = 0), with an arbitrary *F*_1_. Using equation (A 6), we proceed iteratively to (*F*_*n*+1_, *ϕ*_*n*+1_). *ϕ*_*N*_ corresponds to the contact of the *N*th sphere with the wall, which can be made equal to zero (corresponding to *θ*_*N*_ = 0, see figure 12) by adjusting the value of G [22,23].

Compression Δ is defined as Δ = (*N D* − *L*)/*D*, where *L* is the total chain length. This results in

A 7 $$\Delta =N-\sum_{n=1}^{N}\cos\theta_{n}=N-\sum_{n=1}^{N}{\left(1+\phi_{n}^{2}\right)}^{-1/2}.$$

The total energy is given by the sum of the energy due to transverse sphere displacement and the energy due to tilt,

A 8 $$E=\frac{1}{2}\sum_{n=1}^{N}F_{n}^{2}+\tau \left(\sum_{n=0}^{N}(n\cos\theta_{n})-\frac{N}{2}\right)=\frac{1}{2}\sum_{n=1}^{N}F_{n}^{2}+\tau \left(\sum_{n=0}^{N}\frac{n}{\sqrt{1+\phi_{n}^{2}}}-\frac{N}{2}\right),$$

where we have used $\cos\theta_{n}={\left(1+\phi_{n}^{2}\right)}^{-1/2}$. For the case of a straight chain (*F*_*n*_ = 0, *θ*_*n*_ = 0, *ϕ*_*n*_ = 0), this reduces to

A 9 $$E_{s}=\frac{\tau N^{2}}{2}.$$

## Appendix B. Transition to the continuous formulation

In a continuum description both the angle *θ* (or *ϕ*) and the transverse force *F* are functions of a continuous variable *u*. The approximate continuum representation of the iterative relations, equation (A 6), may then be obtained as follows, where for the simplicity of the argument we initially set *τ* = 0.

Using the first of the iterative equations, equation (A 6), we obtain

B 1 $$F_{n}+F_{n+1}=G(\phi_{n+1}+2\phi_{n}+\phi_{n-1}).$$

Re-expressing the left-hand side using the second iterative equation results in

B 2 $$\frac{\phi_{n}}{\sqrt{1+\phi_{n}^{2}}}=G(\phi_{n+1}-2\phi_{n}+\phi_{n-1})+4G\phi_{n}.$$

The term in brackets on the r.h.s. may be identified as a central difference approximation of the second derivative of a continuous function *ϕ*(*u*) with respect to a continuous variable *u*, evaluated at *u* = *n*. A continuum formulation of this equation is thus given by

B 3 $$\phi^{\prime \prime }(u)=-4\phi (u)+\frac{\phi (u)}{G\sqrt{1+\phi^{2}(u)}},$$

G is the compressive force exerted at both ends of the chain (see appendix A).

As in the discrete formulation, the presence of tilt makes the compressive force between contacting spheres a linear function of the sphere number. We thus replace the constant G by

B 4 G(u)=G+τu,

to arrive at

B 5 $$\phi^{\prime \prime }(u)=-4\phi (u)+\frac{\phi (u)}{G(u)\sqrt{1+\phi^{2}(u)}}.$$

We will call equation (B 5) the *full continuum equation with tilt*. This is the differential equation that is the basis of the continuous description used in this article.

## Appendix C. Compression and energy in the continuous formulation

It remains to develop corresponding expressions for compression Δ and energy *E*, for a given profile *ϕ*(*u*). Compression in the discrete representation is given by equation (A 7); in the continuous formulation (with *ϕ* = tan*θ*), this translates into

C 1 $$\Delta =\int_{0}^{N}(1-\cos(\arctan\phi (u)))\;du=\int_{0}^{N}(1-(1+\phi^{2}{\left(u)\right)}^{-1/2})\;du.$$

For *ϕ*(*u*) ≪ 1, consistent with the approximation that is the general basis of the continuous formulation, we obtain

C 2 $$\Delta ≃\frac{1}{2}\int_{0}^{N}\frac{\phi^{2}(u)}{1+\phi^{2}(u)/2}\;du≃\frac{1}{2}\int_{0}^{N}\phi^{2}(u)\;du.$$

An expression for the energy of a solution *ϕ*(*u*) of the continuum equation requires an integral formulation of equation (A 8). Similar to the transition from *ϕ*_*n*_ to *ϕ*(*u*) we introduce a continuous function *F*(*u*) with respect to a continuous variable *u*, which corresponds to *F*_*n*_ when evaluated at *u* = *n*. Using the linear interpolation *F*(*u* + 1/2) ≃ (1/2)(*F*(*u*) + *F*(*u* + 1), together with equation (A 6) we obtain an approximate expression for the displacement *F*(*u* + 1/2) in terms of *ϕ*(*u*),

C 3 $$F^{2}(u+1/2)≃\frac{1}{4}\frac{\phi^{2}(u)}{1+\phi^{2}(u)}.$$

Using the expression for the total energy in the discrete case, equation (A 8), we can write its equivalent for the continuum formulation as

C 4 $$E=\frac{1}{2}\int_{1}^{N}F^{2}(u)\;du+\tau \left(\int_{0}^{N}\frac{u\;du}{\sqrt{1+\phi^{2}(u)}}\right)≃\frac{1}{8}\int_{0}^{N}\frac{\phi^{2}(u)}{1+\phi^{2}(u)}\;du+\tau \left(\int_{0}^{N}\frac{u\;du}{\sqrt{1+\phi^{2}(u)}}\right).$$

We note that for the case of the straight chain (*ϕ*(*u*) = 0 for 0 ≤ *u* ≤ *N*) this expression reduces to *E*_*s*_ = *τN*^2^/2, i.e. the same expression as in the discrete case (equation (A 9)).

For *ϕ*(*u*) ≪ 1, and correct to order *ϕ*^2^, we thus obtain

C 5 $$E≃\frac{\Delta }{4}+\frac{\tau }{2}\left(N^{2}-\int_{0}^{N}u\phi^{2}(u)\;du\right),$$

where we have used equation (C 2) for compression Δ.

If we approximate *ϕ*(*u*) by a triangular profile, equation (C 5) can be evaluated to give the following expression for the energy difference between straight and buckled chain,

C 6 $$E-E_{s}≃\frac{\Delta }{4}(1-2\tau N),$$

to lowest order in compression Δ and tilt *τ*.

## Appendix D. Compression only: properties of Jacobi functions solutions

For values of compression Δ≲0.3 solutions to the full continuum equation are well approximated by scaled Jacobi functions, as discussed in detail in [25]. The following provides a summary, together with two new results related to compression Δ and compressive force G.

For the case of the hard wall boundary conditions considered here, i.e. *ϕ*(0) = *ϕ*(*N*) = 0, the solution of the reduced equation in the absence of tilt (*τ* = 0), equation (3.1), is given in terms of the scaled Jacobi *cn* function as

D 1 $$\phi (u)=\phi_{\max}cn\left(\sqrt{\frac{\left(G^{-1}-4\right)}{\left(2m-1\right)}}(u-N/2)|m\right).$$

The so-called modulus $\sqrt{m}$, is related to the period of the Jacobi functions, with

D 2 $$N=2K(m)\sqrt{\frac{(2m-1)G}{\left(1-4G\right)}},$$

where *K*(*m*) is the complete elliptic integral of the first kind.

The peak value *ϕ*_max_ of the variable *ϕ* (at *u* = *N*/2) is given by

D 3 $$\phi_{\max}=2\sqrt{\frac{m}{\left(2m-1\right)}(1-4G)}\;\mathrm{for}\;0<m\leq 1.$$

(In [25] the above expressions were written in terms of the quantity *κ*^2^, with $\kappa^{2}=G^{-1}-4$).

For small values of compression, we can derive an analytical expression for the variation of *ϕ*_max_ with compression Δ. From equation (C 2), we obtain $\Delta ≃1/2\int_{0}^{N}\phi {\left(u\right)}^{2}\;du$. This integral can be evaluated analytically for our scaled Jacobi, resulting in

D 4 $$\Delta =\phi_{\max}^{2}\frac{N}{2K(m)}\frac{E(m)+(m-1)K(m)}{m},$$

where *K*(*m*) and *E*(*m*) are the complete elliptic integrals of the first and of the second kind, respectively. By combining equations (D 2) and (D 3), we obtain a solution for *m* = 1/2, resulting in

D 5 $$\Delta =\phi_{\max}^{2}N\left(\frac{E(1/2)}{K(1/2)}-1/2\right).$$

A further analytical result is available for the value of the compressive force G at Δ = 0. At zero compression, the amplitude *ϕ*_max_ = 0, requiring the product m(1−4G) to be zero, from equation (D 3). The case G=1/4 corresponds to the limit *N* → ∞, a finite value for *N* thus requires *m* = 0. Using equation (D 2), we obtain

D 6 $$G(\Delta =0)={\left[4-{\left(\frac{\pi }{N}\right)}^{2}\right]}^{-1}.$$

## Appendix E. Relation to the Airy equation

To study the variation of the displacement peak for small compression, Δ, we found it sufficient to consider a linearization of the full equation, equation (B 5), in both *ϕ* and *τ*,

E 1 $$\phi^{\prime \prime }=\left[\frac{1}{G}\left(1-\frac{\tau u}{G}\right)-4\right]\phi .$$

By introducing a linear change of variables from *u* to *x* via

E 2 $$x={\left(\frac{\tau }{G^{2}}\right)}^{-2/3}\left[\frac{1}{G}\left(1-\frac{\tau u}{G}\right)-4\right],$$

and renaming *ϕ* = *y* we can rewrite this linearized reduced equation as

E 3 $$y^{\prime \prime }(x)=xy(x).$$

This is the Airy differential equation which has analytical solutions in terms of the Airy *Ai* and *Bi* functions,

E 4 $$y(x)=c_{1}\mathrm{Ai}(x)+c_{2}\mathrm{Bi}(x),$$

with constants *c*_1_ and *c*_2_.

We want to find a solution *ϕ*(*u*) which is zero at the two endpoints *u* = 0 and *u* = *N*. Expressed in terms of *y*(*x*) this corresponds to (from equation (E 2)) *y*(*x*_1_) = *y*(*x*_2_) = 0, with

E 5 $$x_{1}={\left(\frac{\tau }{G^{2}}\right)}^{-2/3}\left[\frac{1}{G}-4\right]\;\mathrm{and}\;x_{2}={\left(\frac{\tau }{G^{2}}\right)}^{-2/3}\left[\frac{1}{G}\left(1-\frac{\tau N}{G}\right)-4\right].$$

It remains to fix the constants *c*_1_ and *c*_2_ in equation (E 4). From the boundary condition *y*(*x*_1_) = 0, we obtain

E 6 $$\frac{c_{2}}{c_{1}}=-\frac{\text{Ai}(x_{1})}{\text{Bi}(x_{1})}=-\frac{\text{Ai}(x_{2})}{\text{Bi}(x_{2})},$$

and we can thus write the solution *y*(*x*), equation (E 4), as

E 7 $$y(x)=c_{1}\left(\mathrm{Ai}(x)-\frac{\text{Ai}(x_{1})}{\text{Bi}(x_{1})}\mathrm{Bi}(x)\right).$$

The constant *c*_1_ can be expressed in terms of compression, Δ, as follows.

For *ϕ* ≪ 1 compression, Δ, is given by equation (C 2) as

E 8 $$\Delta =\frac{1}{2}\int_{0}^{N}\phi^{2}(u)\;du=f\;(\tau ,G)\int_{x_{1}}^{x_{2}}y{\left(x\right)}^{2}\;dx,$$

with the prefactor $f\;(\tau ,G)=(4-(1/G)(1-(\tau N/G)))/2{\left(\tau /G^{2}\right)}^{1/3}$. For our Airy function solution, equation (E 4), this integral over *y*(*x*)^2^ can be computed analytically to result in

E 9 $$\Delta =f\;(\tau ,G)(y^{\prime }{\left(x_{2}\right)}^{2}-y^{\prime }{\left(x_{1}\right)}^{2}).$$

Compression is thus expressed in terms of the slopes *y*′(*x*) at the two endpoints, *x*_1_, *x*_2_. Using our solution for *y*(*x*), equation (E 7), this results in

E 10 $$\Delta =c_{1}^{2}f\;(\tau ,G)\left[{\left(Ai^{\prime }(x_{2})-\frac{Ai(x_{1})}{Bi(x_{1})}Bi^{\prime }(x_{2})\right)}^{2}-{\left(Ai^{\prime }(x_{1})-\frac{Ai(x_{1})}{Bi(x_{1})}Bi^{\prime }(x_{1})\right)}^{2}\right],$$

and thus provides an expression for the constant *c*_1_ in terms of compression Δ, tilt *τ*, compressive force G and sphere number *N*. We can revert from *y*(*x*) to *ϕ*(*u*) via equation (E 2) and thus have obtained an analytical solution of the linearized reduced equation with tilt, equation (E 1), in terms of these parameters (figure 9). (Note that from experimenting with Mathematica we find that (for given *N*) not all pairs of (τ,G) lead to solutions which fulfill the boundary conditions *y*(*x*_1_) = *y*(*x*_2_) = 0, with *x*_1_, *x*_2_ defined in equation (E 5).)
